# Metachronic malignant transformation of small bowel and rectal endometriosis in the same patient

**DOI:** 10.1186/1477-7819-4-93

**Published:** 2006-12-12

**Authors:** Joaquin Marchena-Gomez, Alicia Conde-Martel, Marion Hemmersbach-Miller, Ana Alonso-Fernandez

**Affiliations:** 1 Department of General Surgery, University Hospital Gran Canaria "Dr. Negrin", Las Palmas G.C., Spain; 2 Department of Internal Medicine, University Hospital Gran Canaria "Dr. Negrin", Las Palmas G.C., Spain; 3 Department of Pathology, University Hospital Gran Canaria "Dr. Negrin", Las Palmas G.C., Spain

## Abstract

**Background:**

Malignant transformation of intestinal endometriosis is a rare event with an unknown rate of incidence. Metachronous progression of endometriosis to adenocarcinoma from two distant intestinal foci happening in the same patient has not been previously reported.

**Case presentation:**

We describe a case of metachronic transformation of ileal and rectal endometriosis into an adenocarcinoma occurring in a 45-year-old female without macroscopic pelvic involvement of her endometriosis. First, a right colectomy was performed due to intestinal obstruction by an ileal mass. Pathological examination revealed an ileal endometrioid adenocarcinoma and contiguous microscopic endometriotic foci. Twenty months later, a rectal mass was discovered. An endoscopic biopsy revealed an adenocarcinoma. En bloc anterior rectum resection, hysterectomy and bilateral salpingectomy were performed. A second endometrioid adenocarcinoma arising from a focus of endometriosis within the wall of the rectum was diagnosed.

**Conclusion:**

Intestinal endometriosis should be considered a premalignant condition in premenopausal women.

## Background

The development of a malignancy is a relatively common complication of endometriosis [1]. In fact, several publications have reported malignant neoplasms arising from endometriosis. Most of these publications are case reports or refer to a small series of patients presenting either ovarian carcinomas with associated endometriosis or invasive endometrioid adenocarcinomas involving adjacent pelvic structures [2]. Malignant transformation of extraovarian endometriosis, including the intestinal tract, however, has not been reported as frequently [3]. The largest reported series of neoplastic changes in gastrointesinal endometriosis includes 17 cases [4] We describe a case of metachronic malignant transformation arising from two different intestinal endometriotic sites, ileal and rectal, occurring in the same patient.

## Case presentation

A 45-year-old woman was admitted to the hospital because of a 5 month history of paroxistic abdominal pain and vomiting. The patient's past medical history included ovarian endometriosis treated with bilateral oophorectomy in another hospital seven years earlier. She subsequently received treatment with medroxyprogesterone and transdermal estrogens.

On physical examination the abdomen was distended and bowel sounds were increased. Laboratory values were unremarkable, and abdominal x-rays confirmed the diagnosis of intestinal obstruction. Laparotomy revealed an ileal mass protruding into the lumen. A right hemicolectomy including 40 cm of ileum was performed. The female genital tract and pelvis had no macroscopic evidence of endometriotic lesions.

**Figure 1:**
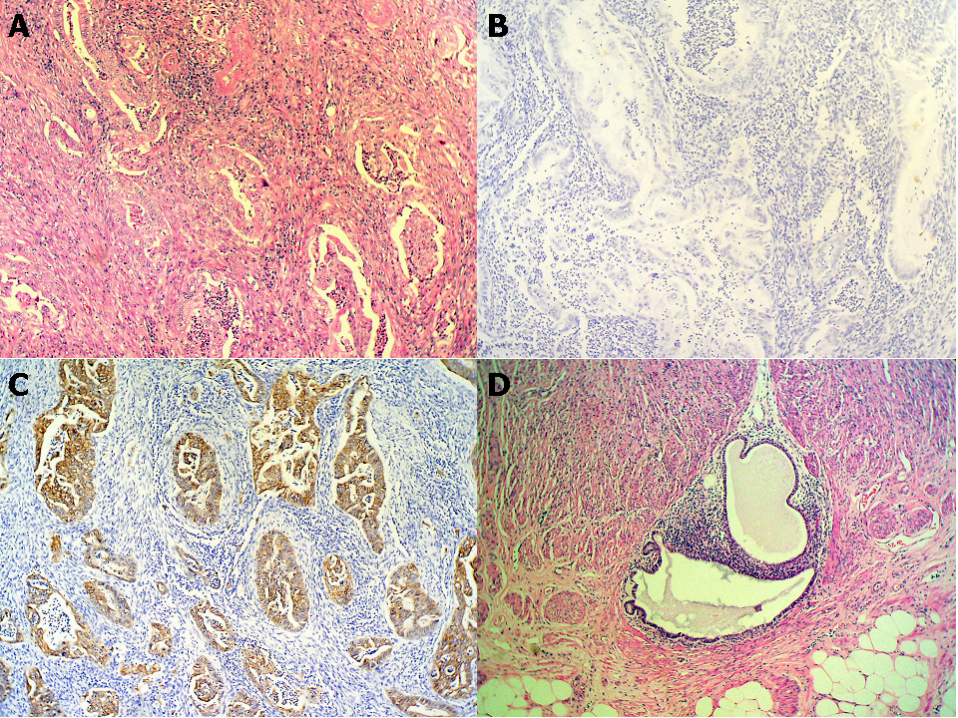
A. Infiltrative endometrioid adenocarcinoma of the ileum (hematoxylin-eosin stain, 100×). B. Cytokeratin 20 immunostaining negative (100×). C. Cytokeratin 7 immunostaining positive (100×). D. Cecum intraparietal benign endometriosis (hematoxylin-eosin stain, 100×).

Histopathology revealed the following: the resection specimen consisted of 16 cm of right colon and 39 cm of ileum. A tumor measuring 5.5 cm was found within the wall of the ileum 2 cm from the ileocaecal valve. Morphologic and immunohistochemical features were typical of an endometrioid adenocarcinoma (cytokeratin (CK) 7-positive, vimentin-positive, CK20-negative) (figure 1). The high nuclear grade tumor involved the mucosa, the muscularis propria and the subserosal fat. Vascular invasion was noted as well as metastatic involvement of 5 out of 40 isolated lymph nodes. Several foci of endometriosis were identified adjacent to this mass and in the caecum. Both, the epithelial and the stromal cells tested positive for estrogen receptors and for CK7, while tests for CK20 proved negative. The tumor itself showed only weak positivity for estrogen receptors.

After surgery the patient received subsequent chemotherapy with 5-fluorouracil and leucovorin. Twenty months later the patient noted rectal bleeding. A colonoscopy discovered a mass at 15 cm from the anal edge. The biopsy showed an adenocarcinoma. A second endometrioid adenocarcinoma was not suspected before surgery.

During laparotomy a tumor in the anterior wall of the rectum was seen. It was fixed to the uterus, occupying the recto-uterine pouch which it seemed to invade. No macroscopic endometrioid foci were seen in the pelvis. En bloc resection including rectum, uterus and the fallopian tubes was performed, as an invasion of the uterus was suspected. Transit restoration was achieved by termino-terminal anastomosis using the EEA-stapler device.

**Figure 2:**
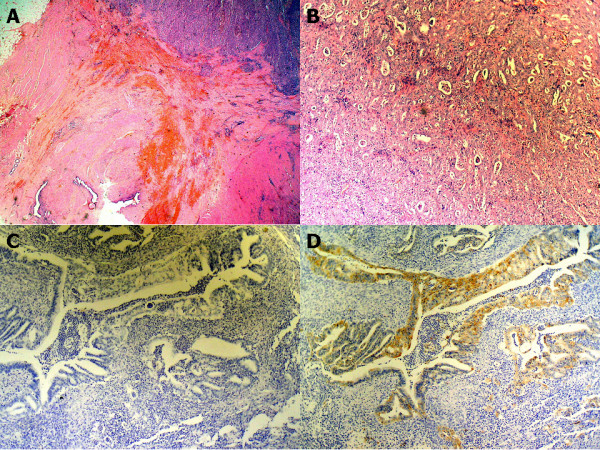
A. Rectal endometrioid adenocarcinoma with adjacent focus of endometriosis (hematoxylin-eosin stain, 20×). B. Rectal endometrioid adenocarcinoma (hematoxylin-eosin stain, 100×). C. Cytokeratin 20 immunostaining negative (100×). D. Cytokeratin 7 immunostaining positive (100×).

Histopathology revealed the following: The resection specimen consisted of 18 cm of rectum, the uterus and the fallopian tubes. Histological examination confirmed the presence of a tumor measuring 3.5 cm in diameter, located in the anterior wall of the rectum and embedded to the posterior wall of the uterus. The poorly differentiated adenocarcinoma involved all layers of the intestine up to the uterus which was not invaded microscopically. The uterus and the fallopian tubes had no major microscopic changes. Morphological and immunohistochemical features of this endometriotic adenocarcinoma were similar to the previously removed tumor (CK7- positive, vimentin-positive, and CK20 negative) (figure 2). There was no lymph node involvement in any of the 17 removed lymph nodes. Multiple microscopic foci of endometriosis were seen, especially in the peritumoral and peritubaric areas. Once again, immunohistochemical features of these foci included positive estrogen receptors and a positive result on CK7 in the epithelial and stromal cells, and negative results for CK20. After the second operation, the patient started treatment with raltitrexed (Tomudex^®^) and 5-Fluorouracil. After five years, she remains asymptomatic and has no evidence of recurrence.

## Discussion

Some studies suggest that the development of malignancies may occur in up to 5.5 % of female patients with endometriosis [1,3]. Only 21.3% of the cases arise from extragonadal pelvic sites, and endometriosis-associated intestinal tumors are even rarer [5]. Endometriosis affects the intestinal tract in 15% to 37% of patients with pelvic endometriosis, most commonly at the rectosigmoid colon followed in frequency by the proximal colon, small intestine, caecum, and appendix [4]. Malignant transformation of primary gastrointestinal endometriosis without pelvic involvement is uncommon, and its real incidence is unknown [1]. It can mimic a primary gastrointestinal neoplasm. Most of these neoplasms are carcinomas, but sarcomas and müllerian adenosarcomas have also been described [3,4,6]. Petersen *et al *[7], in a large review of the previously published endometrioid adenocarcinomas arising in colorectal endometriosis, report less than 50 cases of neoplastic transformation, 22 of which were adenocarcinomas. The others included sarcomas and mixed müllerian tumors.

The progression to invasive cancer has been related with hyperestrogenism, either of endogenous or of exogenous origin [8]. A possible genetic background favoring the onset of cancer has been reported in some patients without hyperestrogenism and with a family history of cancer [9]. The anatomic distribution and frequency of these cancers parallel the occurrence of which benign endometriosis is found at various sites [4].

In order to classify a malignancy as arising from endometriosis, strict histopathologic criteria need to be fulfilled. Sampson [10] first proposed these criteria in the year 1925. He suggested that the following should be fulfilled: 1) the presence of both malignant and benign endometrial tissue in the same organ; 2) the demonstration of cancer arising in the tissue and not invading it from elsewhere; and 3) the finding of tissue resembling endometrial stroma surrounding characteristic glands. Years later, Scott [11] suggested an additional qualification to complete Sampson's criteria: the demonstration of microscopic benign endometriosis contiguous with the malignant tissue. Our patient fulfilled the mentioned criteria in both events. Also, both neoplasms could be considered as having developed from the same tumor, as their immunohistochemical features were the same. Immunohistochemistry is extremely useful for determining the origin of adenocarcinomas. A panel of immunohistochemical stains, including CK 7, CK 20, vimentin, and estrogen receptors, may be used in order to differentiate the colonic adenocarcinoma from the müllerian endometriotic adenocarcinoma [12,13]. Endometrioid adenocarcinoma is usually vimentin-positive. CK 7 is detected in different types of carcinoma, but it is not frequent in the colonic ones, unlike CK 20 which is characteristically from the carcinomas of colonic origin. Both tumors from this patient were vimentin-positive, CK7-positive, and CK20-negative.

Although these immunohistochemical features can also be found in ovarian cancer, we were able to rule out a previous history of malignant ovarian condition in our patient. Seven years earlier, she underwent surgery because of benign endometriosis, and in the surgical procedures that followed, no data of ovarian carcinomatosis was found.

We think that this case demonstrates histological progression from endometriosis to invasive adenocarcinoma involving, metachronically, two different segments of the gastrointestinal tract in the same patient with more than one years' lapse in appearance.

There is no adequate classification to grade and categorize the extension of endometrioid adenocarcinomas of intestinal origin. Classification may be more complicated because these tumors arise from peritoneal implants and invade organs from the outer to the inner layers. Some authors [14] have used the FIGO staging to classify extraovarian carcinomas that arise from endometriosis. Nevertheless, even this classification has been questioned for ovarian tumors arising from endometriosis [15].

For those endometrioid tumors which originate within the wall of the intestine, we suggest using the TNM score for intestinal tumors. Thus, as in this case, the ileal tumor could be classified as T2N2M0 and the rectal one as T3N0M0.

The main interest of this case is that the widespread intraparietal bowel wall endometriosis was discovered without any macroscopic pelvic foci, similar to the case described by Orlandi *et al*.[16]. Different hypothesis trying to explain the appearance of endometrioid tissue at extraovarian sites have been developed. The most prevailing hypothesis proposes that endometriosis results from implantation of endometrial tissue that gains access to the peritoneal cavity by retrograde flow during menstruation, but most likely other factors also have an influence (genetics, immunological factors, environment etc.). Another theory implies the migration of the cells trough the lymphatic system or via hematogenous spread, as might have been the case in our patient.

Recently a new concept in the pathogenesis of endometriosis has been developed: the "neurologic hypothesis". Possover *et al *[17] found that a comparison of the most involved pelvic sites showed an absolute correlation with the anatomical repartition of the pelvic sympathetic nervous system. On the other hand, Anaf *et al *[18] demonstrated that there is a close histological relationship between endometriotic lesions of the large bowel and the nerves of this area. Endometrioid lesions seem to infiltrate the large bowel wall preferentially along the nerves, even at distance from the palpated lesion.

Endometriosis and its possible malignant changes should be taken into account in the differential diagnosis of intestinal masses in females. Also, clinical suspicion for malignancy should be aroused in patients with abdominal pain or rectal bleeding and a previous history of quiescent endometriosis. Recognition of these lesions is important because of the different management required by primary gastrointestinal neoplasms and by those arising from endometriosis. These differences may have significant clinical implications.

Since intestinal resection can be performed safely in most women with endometrioid bowel involvement, some authors strongly support the use of aggressive surgical extirpation of all visible intestinal endometriosis in patients with advanced disease [19]. Total abdominal hysterectomy and bilateral salpingo-oophorectomy at the time of bowel resection correlates with improved outcome [20].

Controversy exists, however, over the optimal adjuvant treatment, particularly in those patients whose disease is completely eliminated. Radiotherapy has been given in some cases [1,4,8,14], especially in patients with limited pelvic involvement. Yantis *et al*. [4] mention that only 3 out of 17 patients underwent radiotherapy, including one with an incomplete surgical resection. In a large series of ovarian and extraovarian endometriosis-associated cancer, only 15 out of 115 patients received radiotherapy [14].

Chemotherapy has been used more frequently, but the value of this treatment is not yet proven [1]. Due to the limited number of cases of malignant transformation of extragonadal endometriosis and the lack of an adequate classification, conclusions about the utility of chemotherapy are only drawn from studies that also include malignant ovarian endometriosis. The most used drugs for chemotherapy in the last years have been platinum in combination with paclitaxel [14]. After consulting with the oncology department, our patient was treated as if both events were a primary intestinal adenocarcinoma. This kind of treatment is debatable, but the patient remains asymptomatic five years after her second tumor had been extirpated.

Globally, this condition is very rare. Thus, is it really appropriate to speak about "premalignancy"? Should endometriosis be considered a premalignant condition? Considerable conflict exists in the literature regarding the relationship between endometriosis and cancer [21]. Most likely, ovarian endometriosis "per se" should not be considered a premalignant condition if reviewing the available literature. But we do support the idea that this situation is very different if we are considering endometriosis localized in the intestinal wall. The presented case supports this statement.

## Conclusion

This case is, to our knowledge, the first report on metachronic malignant transformation of intestinal endometriosis from two distant intestinal foci. Intestinal endometriosis should be considered a premalignant condition.

## Competing interests

The author(s) declare that they have no competing interests.

## Authors' contributions

**JMG **conceived the study, participated in its design and coordination and helped to draft the manuscript.

**ACM **participated in the sequence alignment and drafted the manuscript.

**MHM **participated in the sequence alignment and translated the manuscript.

**AAF **carried out the histopathology studies

All authors read and approved the final manuscript
